# Environmental Characteristics Associated with Older Adults’ Social Participation: The Contribution of Sociodemography and Transportation in Metropolitan, Urban, and Rural Areas

**DOI:** 10.3390/ijerph17228399

**Published:** 2020-11-13

**Authors:** Mélanie Levasseur, Daniel Naud, Jean-François Bruneau, Mélissa Généreux

**Affiliations:** 1 School of Rehabilitation, Faculty of Medicine and Health Sciences, Pavillon Gérald-Lasalle, Université de Sherbrooke, Sherbrooke, QC J1H 5N4, Canada; 2 Research Centre on Aging, Estrie Integrated University Health and Social Services Centre—Sherbrooke Hospital University Centre, Sherbrooke, QC J1H 4C4, Canada; Daniel.Naud2@USherbrooke.ca; 3 Interuniversity Research Centre on Enterprise Networks, Logistics and Transportation, Montreal, QC G1V 0A6, Canada; Jean-francois.bruneau@cirrelt.ca; 4 Department of Community Health Sciences, Université de Sherbrooke, Sherbrooke, QC J1H 5N4, Canada; Melissa.Genereux@USherbrooke.ca

**Keywords:** population health, transportation, paratransit, regression, monthly social engagements, local environment

## Abstract

Although social participation fosters older adults’ health, little is known about which environmental characteristics are related to greater participation in social activities. The Canadian Community Health Survey (*n* = 2737), a transportation survey, and multiple secondary data sources were used to identify the environmental characteristics associated with older Quebecers’ social participation according to living area. Greater social participation was associated with: (1) a higher concentration of older adults (IRR = 2.172 (95% CI 1.600, 2.948); *p* < 0.001), more kilometers traveled by paratransit (IRR = 1.714 (95% CI 1.286, 2.285); *p* < 0.01), a lack of medical clinics (IRR = 0.730 (95% CI 0.574, 0.930); *p* = 0.01), and more funded home adaptations (IRR = 1.170 (95% CI 1.036, 1.320); *p* = 0.01) in large metropolitan areas; (2) larger paratransit fleets (IRR = 1.368 (95% CI 1.044, 1.791); *p* = 0.02) and a lower density of road intersections (IRR = 0.862 (95% CI 0.756, 0.982); *p* = 0.03) in regular metropolitan areas; (3) less social deprivation (IRR = 1.162 (95% CI 1.025, 1.318); *p* = 0.02) in urban areas; and (4) a higher concentration of older populations (IRR = 2.386 (95% CI 1.817, 3.133); *p* < 0.001) in rural areas. According to these findings, social participation interventions should target the local environment—for example, by providing more social interaction opportunities for older adults living in younger neighborhoods and by improving access to public transportation, especially paratransit.

## 1. Introduction

Population aging is a demographic challenge that requires effective and innovative interventions to improve population health and well-being. Adults aged 65 and over make up a growing percentage of the population (16.9% in Quebec in 2016; Statistics Canada, 2016), and the World Health Organization (WHO) estimates that this percentage will almost double by the middle of this century (WHO, 2015). Considering that most Quebecers are aging in their homes, this demographic change has significant consequences for individuals, their communities, and health and social services [1]. Many older adults report living with chronic illnesses, and almost half have or will have disabilities [2]. However, chronic diseases and disabilities can be prevented or mitigated by interventions targeting the social determinants of health, such as social participation [3]. Additionally, the coronavirus (COVID-19) has caused a high mortality rate amongst older adults (including community-dwelling seniors) and, following self-isolation recommendations, many of them continue to engage in avoidance behaviors, which could be detrimental to their physical and mental health [4].

Social participation, which is regarded as an important dimension of active aging, especially because it helps older adults to stay integrated in their community, is associated with many health outcomes [5]. Social participation is defined as a person’s involvement in activities that provide social interactions in the community [6]. Greater social participation is associated with fewer disabilities [7] and depressive symptoms [8], preserved cognitive functions [9], and shorter hospital stays [10]. A recent meta-analysis showed that stronger social relationships were associated with a 50.0% greater likelihood of survival than weaker relationships, a protective effect comparable to quitting smoking or avoiding other recognized risk factors [11].

Measures or interventions targeting the physical and social characteristics of the environment can facilitate social participation [12]. **Physical characteristics** that positively influence social participation include reliable and accessible transportation options and the availability of resources and activities. For instance, having a driver’s license and access to a car provides greater independence for social activities, especially when health and mobility are declining [13]. In metropolitan areas, the use of public transit is associated with greater social participation [14]. Moreover, door-to-door paratransit improves the social participation of older adults who have disabilities and facilitates their mobility and community integration [15]. Moreover, low traffic density and traffic safety were shown to be associated with greater participation in the community [16], as were pedestrian-oriented neighborhoods [17]. By facilitating formal and informal social interactions, the availability of resources and activities (restaurants, stores, sports centers, etc.) is also associated with social participation [14]. The available resources (health services, public furniture, walking trails, etc.) must nevertheless be convenient for older adults [18].

**Social characteristics** of the environment, such as a larger population size [19] and a neighborhood’s affluence and perceived safety [18], are also associated with greater social participation. For example, social cohesion, which encompasses notions of a shared value system, trust, and reciprocity, facilitates social participation through a network of opportunities and social connections [13]. Living areas—i.e., metropolitan, urban, and rural—which are defined by different characteristics and living experiences, were found to be characterized by different health levels in the province of Quebec, Canada [20]. In addition, the COVID-19 pandemic restrained older adults from making social contacts, discouraged them from engaging in community activities, and infused ageist discourses, generating fear, anxiety, and stigma that may further limit their social participation [21]. The health and well-being of Quebec’s aging population could benefit from local interventions facilitating mobility and informal social contacts, and improving access to resources and activities, especially in the current context of the pandemic. It has been, and still is, important to take living area and environmental characteristics into account to foster active aging and older adults’ social participation [14].

### Social Participation According to Living Area in Quebec

A recent study showed that Quebec older adults participated on average in one social activity every other day, the lowest participation rate across all Canadian provinces [22]. Frequency of social participation was also found to be similar according to living area but specific activities differed; for example, sports and cultural events were more frequent in metropolitan than urban or rural areas [14]. Moreover, a higher frequency of social participation was associated with the perception of shorter walking times to neighborhood resources in metropolitan and urban areas and better access to resources in rural areas [14]. Another study conducted in Quebec did not find any differences in either social participation or factors associated with participation according to older adults’ living area [23]. A larger metropolitan area such as Montreal is, however, taken as the only metropolitan region [23] or the sole focus [24,25,26,27] in some studies. Social participation studies should include and compare other metropolitan areas with Montreal. A better understanding of the environmental characteristics that influence social participation in Quebec is lacking, including large (>2 M inhabitants) and regular metropolitan, urban, and rural areas [28]. This study thus aimed to identify the environmental characteristics associated with older Quebecers’ social participation, according to large metropolitan (i.e., Montreal), regular metropolitan, urban, and rural areas, while controlling for individual characteristics.

## 2. Materials and Methods

### 2.1. Design and Participants

**Figure 1:**
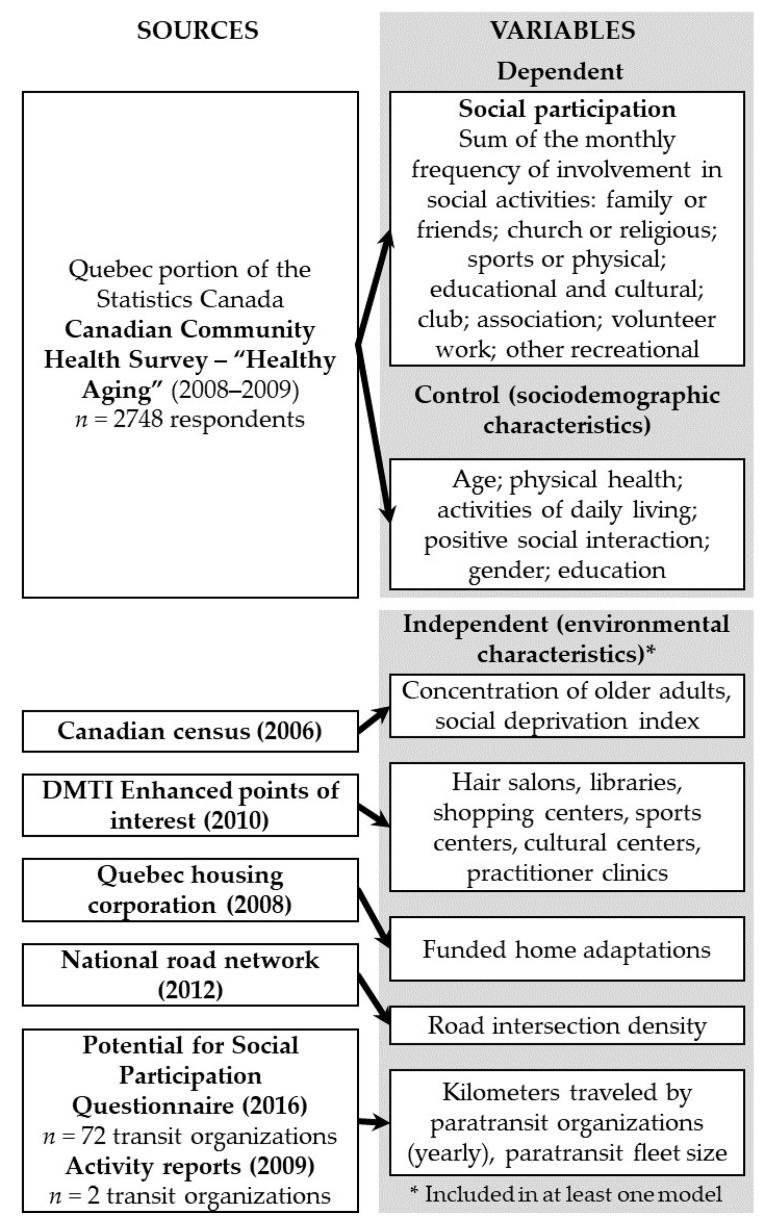
Diagram of data sources and selected variables.

This study is part of a larger research program aimed at developing a decision-support tool, in the form of an online interactive atlas, that represents the potential for social participation at the neighborhood level [29]. As part of the research program, a scoping study [30,31] determined the environmental indicators associated with older adults’ social participation, and two surveys were conducted to characterize: (1) their perception of the indicators [29] and (2) regular transit and paratransit services according to living area [32]. The current study combined individual- and environmental-level datasets collected from a survey and secondary sources (Figure 1). We first used individual microdata drawn from the Quebec sample—i.e., 2748 respondents aged 65 and over living in private dwellings—in the cross-sectional 2008–2009 Canadian Community Health Survey “Healthy Aging” (CCHS-HA) to conduct secondary analyses. At the time of the study, only the CCHS-HA included questions about the social participation of older adults. It should be noted that the Canadian Longitudinal Study on Aging (CLSA) [33] data are now available and could be used in future studies to replicate the current results. The CCHS-HA respondents were interviewed in person and recruited using a stratified random sampling strategy based on age, gender, and rural or urban areas [34]. Full-time members of the Canadian Forces and residents of the three territories—Indian reserves, Crown lands, and some remote regions—representing about 4% of the target population, were excluded from the sampling.

The environmental characteristics found to be associated with social participation were selected based on the results of a scoping study [31] and its update [30] according to the International Classification of Functioning, Disability, and Health (ICF) [35]. To collect data based on this comprehensive definition of the environment, multiple sources were reviewed according to their quality, spatial coverage, and scale (Table S1 and Figure 1). Finally, all regular transit and paratransit organizations (*n* = 164) in Quebec listed by the Ministry of Transportation were surveyed during the summer of 2017 [36]. 

Environmental characteristics were paired with the individual respondents using Statistics Canada’s dissemination area (DA) unique identification. The DA is the smallest standard geographic area available across the country, roughly comparable to a city block or neighborhood [37]. Because the population and characteristics of boroughs are generally more homogeneous than those of census subdivisions (CSD), the former were used in the eight municipalities that had such boundaries. The study was approved by the Research Ethics Committee of the Estrie Integrated University Health and Social Services Centre—the Sherbrooke University Hospital Centre (#2105-465)—while the Statistics Canada Executive Management Board, acting as the Research Ethics Board, approved the CCHS-HA.

### 2.2. Variables

**Social participation**. The dependent variable was measured from the self-reported frequency of involvement in eight activities with others: family or friends outside the household; church or religious; sports or physical; educational and cultural; service club or fraternal organization; neighborhood, community, or professional association; volunteer or charity work; and other recreational activities (e.g., hobbies, bingo, and other games). Based on previous studies, responses were converted into monthly frequency of engagement for each activity [14,27,38,39]. Total score is the sum of the frequencies of the eight activities, where a higher score means more community activities per month. The consistency of the scale was satisfactory (Cronbach’s alpha = 0.72). 

**Neighborhood characteristics**. We collected 49 environmental variables (continuous or discrete), classified in three of the ICF environmental domains (18 variables in *products and technology*; 15 variables in *natural environment and human-made changes to environment*; 16 variables in *services*, *systems, and policies*); they are listed in Table S1, along with the collection year and sources. To better reflect the respondents’ environmental context, the data collected were linked as closely as possible to the CCHS-HA collection period (2008 and 2009). For the Desktop Mapping Technologies Inc. (DMTI) data (Table S1), the services and businesses were extracted using the four-digit Standard Industrial Classification (SIC). When no SIC code was available for a specific category (cultural centers, for example), a list of keywords was queried.

The lack of province-wide data on regular transit and paratransit prompted an original survey to complement the secondary data sources. Regular transit and paratransit organizations were invited to complete a bilingual online Potential for Social Participation Questionnaire (PSPQ), which included 46 questions about regional and municipal transportation organized in five sections: geography and respondents (8 questions), regular transit (10 questions), paratransit (6 questions), subway (11 questions), and commuter train (11 questions) [32,36]. The questions concerned bus stops/metro stations, access to bus and metro, kilometers traveled, reduced fares for older adults, and fleet size. Paratransit is distinguished from regular public transit by its door-to-door, flexible service, generally using minibuses or taxis, and requiring a reservation and eligibility [40]. Eligibility for paratransit is determined by the disability or mobility restrictions that prevent a user from using regular public transit [41]. Validated by five experts and pretested with one public organization offering regular transit and paratransit services, this questionnaire has good face and content validity, as well as good internal consistency (Cronbach’s alpha = 0.77 and 0.75, respectively, for regular transit and paratransit). The transit organizations’ data collection unit was the territory they served, which covers multiple municipalities. To consider the heterogeneity of the municipalities within a territory served, we used a disaggregation method to estimate the transit organizations’ answers on the scale of the CSD. Because the CSD boundaries are perfectly nested within the territory served, the original scale was transformed using a simple areal weighting [42] according to potential ridership and the population aged 65 or over (and according to kilometers of roads for variables related to distances).

**Sociodemographic characteristics**. Self-report answers described the participants’ sociodemographic characteristics: (1) age (in years); (2) self-rated health (0 (poor) to 4 (excellent)); (3) activities of daily living 1 (total impairment) to 5 (no impairment), derived from Fillenbaum [43]; (4) positive social interactions (0 (low) to 16 (high), [44]); (5) gender (man, woman); and (6) education (no diploma, high school diploma, postsecondary diploma).

### 2.3. Statistical Analysis

Environmental variables are described by mean, standard deviation, and range (Table S1) and ordered by ICF domains. Counts were made in both neighborhood (DA) and municipality (CSD) to consider sparser resources provided at the municipal rather than the neighborhood level (e.g., libraries). In addition, before being introduced to the models, counts were standardized by the population aged 65 and over or by the area in square kilometers of the CSD or DA.

We classified respondents across four living areas, based on the DA they lived in: (1) large metropolitan (census metropolitan area (CMA)); (2) regular metropolitan (regular CMA); (3) urban (census agglomeration (CA)); and (4) rural (metropolitan-influenced zone (MIZ)) [28,45]. For the descriptive statistics (Table 1), analyses were conducted for each living area as well as for all respondents. Respondents are described by mean and standard deviation or percentage, according to the type of variable (continuous or categorical, respectively), with 95% confidence intervals. Living areas were compared pairwise using the Bonferroni-adjusted Wald test. Considering its distribution, social participation was modeled and compared with a negative binomial regression [46]. Unlike linear regression, the negative binomial model estimates the incidence rate ratio (IRR), which represents the proportional increase (if exp(b) > 1.00) or decrease (if exp(b) < 1.00) in average monthly social participation associated with a unit increase in an environmental variable. Additionally, even if the respondents were nested in DA and CSD, we did not employ multilevel modeling for two reasons: (1) the complex survey design used for the CCHS is currently incompatible with the required bootstrap resampling for variance calculations [47]; (2) low intraclass correlations do not justify the analysis of nested data [48], which was the case in the current study with the low correlation between respondents in the municipalities (intraclass correlation = 0.02). 

The bivariate associations between social participation and the normalized environmental variables (mean = 0 and standard deviation = 1) with a *p*-value equal to or less than 0.25 were first identified. Following the identification of associated variables, multiple regressions were performed in three steps: (1) the main effect of the identified environmental characteristics was tested by adding them in blocks defined according to ICF domains, ordered by their lowest *p*-values; (2) interactions between independent variables and curvilinearity were tested (labelled as Model 1 in Table 2, Table 3, Table 4 and Table 5); and (3) sociodemographic characteristics (age, physical health, activities of daily living, positive social interaction, gender, and education) were added to control for confounding effects (labelled as Model 2 in Table 2, Table 3, Table 4 and Table 5). Unlike linear regression and as presented for each model, McFadden’s pseudo R^2^ is a relative measure used for fitting the overall model rather than a measure of the dependent variable’s variance explained by the model [49]. To consider the assumptions of the negative binomial distribution, outliers in the social participation variable were identified and removed (*n* = 34) using the *nb_adjust* module in Stata [50]. For data only available on a different scale (i.e., census subdivision (CSD, fitting municipal boundaries), 6-digit postal zones or police service territory), values were transposed to the DA scale. Survey weights were considered in the descriptive statistics and the negative binomial regression, making the sample representative of the Quebec population aged 65 and over [34]. Data were accessed through the Quebec Interuniversity Centre for Social Statistics (QICSS), and all the statistical analyses were carried out using Stata 14.2 (StataCorp LLC, College Station, United States) [51]. 

## 3. Results

Aged between 65 and 104 years old, the respondents participated in approximately one activity every other day. Compared to respondents living in other areas, urban respondents did four more activities per month on average (Table 1). Most participants had many positive social interactions and were not impaired in their activities of daily living. Few respondents had mobility restrictions, but moderately greater mobility was observed in urban than in large metropolitan areas. Respondents generally reported good mental health, but this was slightly better in regular metropolitan areas. Similarly, respondents had good physical health, although it was better in large metropolitan than in rural areas (Table 1). A larger percentage of respondents—i.e., almost four out of five—owned their homes in rural areas, compared to about three out of five in large and regular metropolitan areas. Most lived in a household above the 2009 poverty cut-off, set at CAN$13,551 for a one-person household, plus CAN$5421 per additional adult [52]. The majority lived as a couple but fewer did so in large metropolitan areas. More than a third had a postsecondary degree, except in rural areas, where about a quarter had one. While the majority had a driver’s license (71.0%), less than two out of three in Montreal had one. Finally, the percentage of immigrants was more than six times higher in Montreal than in the other areas (Table 1).

Controlling for individual characteristics, several physical and social environmental characteristics were associated with greater social participation. In large metropolitan areas, associations were found between social participation and the concentration of older adults, kilometers traveled by paratransit organizations, medical clinics, and the number of funded home adaptations (Table 2). Greater social participation was associated with a higher concentration in the neighborhood of the municipality’s older population. The social participation of respondents living in neighborhoods with the highest concentration of older adults (fourth quartile) was more than twice that in neighborhoods with the lowest concentration (first quartile). Greater social participation was also associated with more kilometers traveled by paratransit organizations; however, this association was stronger for the first quartile (Table 2). A higher density of funded home adaptations was weakly associated with greater social participation. Areas with a general practitioner in the neighborhood had less social participation (27.0%). In regular metropolitan areas, greater social participation was associated with a larger paratransit fleet and fewer road intersections (Table 3). In urban areas, less social deprivation was associated with greater social participation, but the interaction factor suggests that social participation could increase with a higher rate of adapted vehicles operated by paratransit organizations (Table 4). In rural areas, greater social participation was associated with higher quartiles of older population concentration in the neighborhood and a lower density of libraries (Table 5).

## 4. Discussion

This study aimed to identify the environmental characteristics associated with older adults’ social participation according to their living area. The characteristics related to population and transportation had the greatest association with social participation. For example, greater social participation was linked to an older population concentration in large metropolitan and rural areas. More paratransit services in large and regular metropolitan areas were associated with further social participation. Furthermore, the COVID-19 pandemic has highlighted the need to develop strategies to support older adults, who may be worried by physical distancing rules, in maintaining their social participation and social networks. The CCHS-HA surveyed Canadians in 2008 and 2009; this might not, however, reflect the social participation of current aging generations. Indeed, younger Canadian aging cohorts have higher education levels than older cohorts [53], which may contribute to increased social participation [54]. The median retirement age of Canadians also rose between 2008 and 2018 [55]; this meant more income and greater access to a car, both of which are associated with greater social participation [13]. However, late retirees rated their health as poorer than earlier retirees [56], which might impede their social participation [57]. Additionally, age-friendly policies were recently adopted by the provincial government [1,58], which may have facilitated older adults’ social participation. While the environmental context may also have changed, notably with respect to COVID-19 countermeasures [21], the present results still indicate ways to foster social participation. While many communities have or will adopt a policy encouraging the participation of older adults, it is important that the modifiable aspects facilitating social activities are clearly understood.

Environmental characteristics differed across living areas, showing varying influences on older populations’ participation. These differences suggest that environmental actions, transformations and policies should be considered at the local or regional level. National programs could address the issue of social participation through mass interventions reducing socioeconomic inequities. The results of this study provide ideas that are suitable for the community, easy to implement, and well within the purview of local decision-makers [59]. For example, although not specifically for the older population, one meta-analysis found that design and land-use policies at the community and street levels increased physical activities in urban and metropolitan areas [60]. Such local interventions on social capital are within the purview of local health and community professionals and could be more affordable than direct interventions on social determinants, such as income disparities.

### 4.1. Living Area’s Age Composition

According to a study by Fisher et al. [61], the association between older population concentration and social participation may be attributable to a larger senior population improving social cohesion (β = 0.295; *p* < 0.05) and providing additional social opportunities with peers. Another study found that older Montrealers in neighborhoods with a younger population were less likely to visit the local park than older adults in census tracts with a higher percentage of seniors [62]. Additionally, older adults living in more affluent neighborhoods were less likely to report lower potential for social participation than those living in less affluent neighborhoods (15 vs. 27%; *p* < 0.01) [13]. Paying closer attention to potential inequalities in social opportunities for older adults living in younger neighborhoods could mitigate the negative influence of a decrease in social cohesion that may occur in the context of the pandemic.

### 4.2. Transportation

The associations between paratransit and social participation found in the current study are not consistent with the literature. According to a study by Dahan-Oliel et al. [63], older Montrealers who used paratransit were least able to maintain and participate in social relationships compared to drivers, walkers, and those using public transit, but the number of participants in that study using paratransit was small (*n* = 5). Additionally, although we found that longer distances traveled by paratransit organizations per kilometer of roads in Montreal was associated with social participation, the number of older adults using paratransit was low. An analysis of a Montreal origin–destination survey showed that paratransit represented less than one percent of all travel modes used by adults aged 50 and over but increased to 2.5% past 80 years old [64]. Older non-drivers mostly use paratransit when informal support, such as friends or family, is not available [65]. However, when drivers stop driving, alternative transportation modes need to be available, since they could otherwise be at risk of social isolation by limiting their non-essential trips [66].

In regular metropolitan areas, a lower road intersection density was found to be associated with less participation. These results might be linked to poorly adapted intersections that do not allow enough crossing time for pedestrians, especially when mobility is declining, which can cause insecurities [67]. For older drivers, road intersections present a greater risk of collisions, which may also limit their out-of-home activities [68].

In urban and rural areas, most older adults have a driver’s license, which, coupled with the lack of alternative transportation options, could explain why no transportation variables were associated with participation. However, another study carried out with CCHS respondents found that over 15% of all women in rural Canada were constrained by transportation problems [22]. According to the CCHS analysis performed by Turcotte [69], outside metropolitan areas half of older Canadians who needed help to get to places outside walking distance reported that they did not use paratransit because this service was not available [69].

### 4.3. Strengths and Weaknesses

This cross-sectional analysis shed light on the characteristics associated with social participation according to living area. The analysis tested the associations between social participation and a comprehensive list of environmental characteristics drawn from several domains, derived from an updated scoping study [30,31]. Using a four-group classification for living area enabled us to analyze large and regular metropolitan areas separately.

Nevertheless, this study has some limitations. Because the social context has changed in many ways since the data for this study were collected (2008-09), especially with the COVID-19 pandemic, the results require confirmation in future studies. It was not possible to include a qualitative assessment of environmental characteristics, which have been shown to be relevant to participation levels [30,70]. Because some characteristics were collected or only available on the scale of the municipality (CSD) rather than the neighborhood (DA), they raise the modifiable areal unit problem and may not necessarily reflect the cohesiveness and homogeneity of the neighborhood [71]. Future research considerations include intra-metropolitan variation, even though no systematic and consensus-based definition of Canadian periurbanity or suburbanity was available at the time of the analysis [72].

## 5. Conclusions

This study modeled older adults’ social participation with a comprehensive list of environmental variables in metropolitan Montreal and regular metropolitan, urban, and rural living areas. Large metropolitan and rural areas with a higher concentration of older population were associated with greater social participation, suggesting that neighborhoods with a lower concentration lacked the resources or cohesiveness that create opportunities for social activities. The availability of public transportation, especially paratransit, fostered social participation in metropolitan areas. The findings suggest that public health interventions designed to increase social participation should be rooted in the local environment. 

## Figures and Tables

**Table 1:** Respondents’ characteristics.

Variable	Quebec *n* = 2748	Large Metro *n* = 1283	Regular Metro *n* = 440	Urban *n* = 426	Rural *n* = 599	
Continuous Variables						
	Mean (SD)	Mean (SD)	Mean (SD)	Mean (SD)	Mean (SD)	*p*-Value ^1^
	[95% CI]	[95% CI]	[95% CI]	[95% CI]	[95% CI]	
Age (years)	74.3 (8.8)	74.5 ^a^ (9.1)	74.6 ^a^ (8.9)	73.5 ^a^ (8.8)	74.0 ^a^ (8.1)	0.18
	[74.1, 74.4]	[74.1, 75.0]	[73.8, 75.4]	[72.8, 74.3]	[73.4, 74.6]	
Social participation (number of community activities/month)	14.9 (17.5)	14.0 ^a^ (18.5)	14.9 ^ab^ (16.1)	18.4 ^b^ (19.7)	14.0 ^a^ (14.4)	<0.01
	[14.0; 15.7]	[11.3, 17.4]	[12.0, 18.6]	[14.8; 23.0]	[13.0, 15.2]	
Positive social interaction [0 (low)–16 (high)]	13.2 (4.7)	13.0 ^a^ (4.9)	13.3 ^a^ (4.9)	13.5 ^a^ (4.4)	13.5 ^a^ (4.4)	0.13
	[13.1, 13.4]	[12.7, 13.3]	[12.9, 13.8]	[13.1, 13.9]	[13.1, 13.9]	
Activities of Daily Living (1 [total]–5 [no impairment])	4.7 (0.9)	4.6 ^a^ (1.0)	4.7 ^a^ (0.8)	4.7 ^a^ (0.8)	4.6 ^a^ (0.9)	0.17
	[4.4, 4.9]	[4.6, 4.7]	[4.6, 4.8]	[4.7, 4.8]	[4.6, 4.7]	
Mobility [1 (cannot walk)–6 (no restriction)]	5.7 (1.1)	5.7 ^ab^ (1.2)	5.7 ^a^ (1.2)	5.8 ^ab^ (0.9)	5.7 ^ab^ (1.0)	<0.05
	[5.7, 5.8]	[5.6, 5.7]	[5.6, 5.8]	[5.7, 5.9]	[5.6, 5.8]	
Mental health (0 [poor]–4 [excellent])	3.1 (1.2)	3.1 ^a^ (1.2)	3.2 ^b^ (1.1)	3.0 ^a^ (1.2)	3.0 ^a^ (1.1)	<0.05
	[3.0, 3.1]	[3.0, 3.2]	[3.1, 3.3]	[2.9, 3.2]	[2.9, 3.1]	
Physical health (0 [poor]–4 [excellent])	3.3 (1.3)	3.4 ^b^ (1.4)	3.2 ^ab^ (1.3)	3.3 ^ab^ (1.3)	3.2 ^a^ (1.2)	0.05
	[3.3, 3.4]	[3.3, 3.5]	[3.1, 3.4]	[3.2, 3.5]	[3.1, 3.3]	
**Categorical Variables**						
	**Percentage**	**Percentage**	**Percentage**	**Percentage**	**Percentage**	***p*-Value ^2^**
	**[95% CI]**	**[95% CI]**	**[95% CI]**	**[95% CI]**	**[95% CI]**	
Homeowner	66.5	60.8 ^b^	64.2 ^b^	66.7 ^ab^	78.6 ^a^	<0.001
	[63.1, 69.9]	[55.6, 66.0]	[54.5, 73.9]	[56.6, 76.8]	[73.8, 83.5]	
Income < low income cut-off (yes)	12.0	15.4	8.9	9.9	15.4	0.13
	[9.9, 14.2]	[8.1, 16.4]	[6.1, 11.8]	[5.8, 14.0]	[10.6, 20.2]	
In a couple	60.7	56.8 ^b^	62.6 ^ab^	64.4 ^ab^	64.6 ^a^	<0.05
	[58.4, 63.0]	[53.0, 60.5]	[56.5, 68.7]	[57.7, 71.0]	[60.6, 68.6]	
Retired	94.2	94.0 ^a^	94.7 ^a^	94.3 ^a^	94.2 ^a^	0.99
	[92.8, 95.5]	[91.8, 96.2]	[91.8, 97.6]	[90.8, 97.7]	[91.8, 96.5]	
[Education] No diploma	54.7	52.0 ^b^	48.2 ^b^	53.6 ^b^	64.6 ^a^	0.001
	[51.5, 57.8]	[47.4, 56.6]	[40.5, 56.0]	[46.4, 60.8]	[59.3, 69.8]	
[Education] High school diploma	10.9	12.1 ^a^	12.5 ^a^	10.2 ^a^	7.8 ^a^	0.17
	[9.1, 12.7]	[9.5, 14.8]	[7.5, 17.5]	[6.0, 14.4]	[4.9, 10.8]	
[Education] Postsecondary diploma	34.5	35.9 ^b^	39.3 ^b^	36.2 ^b^	27.6 ^a^	<0.05
	[31.9, 37.1]	[31.8, 39.9]	[32.4, 46.1]	[29.3, 43.2]	[23.2, 31.9]	
Has a driver’s license	71.0	63.1 ^b^	77.0 ^a^	80.7 ^a^	76.2 ^a^	<0.001
	[68.7, 73.4]	[58.7, 67.5]	[71.7, 82.3]	[76.3, 85.0]	[72.5, 80.0]	
Immigrant	11.8	22.2 ^b^	3.3 ^a^	3.2 ^a^	3.0 ^a^	<0.001
	[9.2, 14.4]	[17.5, 26.9]	[1.3, 5.3]	[0.2, 6.2]	[1.2, 4.8]	

^1^ ANOVA; ^2^ Chi^2^; ^a,b^ living areas sharing a superscript letter are not statistically different after Bonferroni correction (*p* > 0.05).

**Table 2:** Association of environmental and individual characteristics with social participation in large metropolitan areas.

Variable	Model 1 (*n* = 1184)			Model 2 (*n* = 1097)		
	exp(b)	exp(b) 95% CI		exp(b)	exp(b) 95% CI	
Concentration of older adults ^#^ (vs. Quartile 1 (Q1))						
Q2	1.075	0.892	1.295	1.071	0.897	1.278
Q3	1.530 ***	1.227	1.909	1.411 **	1.129	1.765
Q4	2.272 ***	1.600	3.226	2.172 ***	1.600	2.948
Km traveled by paratransit organizations ^†^ (CSD) (vs. Q1)						
Q2	1.729 ***	1.330	2.246	1.744 ***	1.349	2.255
Q3	1.514 **	1.142	2.008	1.504 ***	1.139	1.987
Q4	1.585 **	1.193	2.107	1.714 ***	1.286	2.285
Hair salons/km^2^ (DA) (vs. Q1) Q2	1.130	0.990	1.289			
At least one general practitioner clinic (DA)	0.698 **	0.563	0.866	0.730 *	0.574	0.930
Funded home adaptations/km^2^ (CSD)	1.208 **	1.070	1.365	1.170 *	1.036	1.320
(Funded home adaptations/km^2^) squared	0.960 *	0.929	0.991	0.972	0.940	1.005
Age				1.001	0.992	1.009
Physical health				1.065 *	1.002	1.131
Activities of daily living				1.213 **	1.083	1.360
Positive social interaction				1.058 ***	1.041	1.074
Man (vs. woman)				0.905	0.784	1.046
Education (vs. no diploma)						
High school diploma				1.084	0.882	1.333
Postsecondary diploma				1.135	0.989	1.301
McFadden’s pseudo R^2^	0.011			0.025		
Prob > F	<0.001			<0.001		

* 0.05 < *p* < 0.01; ** 0.01 < *p* < 0.001; *** *p* < 0.001. Original variable names: ^#^ 2006 population aged 65 and over (dissemination area (DA))/2006 population aged 65 and over (census subdivision (CSD)); ^†^ km traveled (paratransit)/roads (km).

**Table 3:** Association of environmental and individual characteristics with social participation in regular metropolitan areas.

Variable	Model 1 (*n* = 436)			Model 2 (*n* = 403)		
	exp(b)	exp(b) 95% CI		exp(b)	exp(b) 95% CI	
Paratransit fleet size ^#^ (CSD)	1.394 **	1.137	1.710	1.368 *	1.044	1.791
Paratransit fleet size ^#^ squared	0.866 **	0.795	0.944	0.866 *	0.775	0.969
Road intersection density (DA)	0.835 **	0.749	0.931	0.862 *	0.756	0.982
Age				0.995	0.982	1.009
Physical health				0.976	0.887	1.075
Activities of daily living				1.237	0.994	1.539
Positive social interaction				1.042 **	1.017	1.068
Man (vs. woman)				0.921	0.772	1.100
Education (vs. no diploma)						
High school diploma				1.188	0.830	1.702
Postsecondary diploma				1.050	0.894	1.234
McFadden’s pseudo R^2^	0.002			0.015		
Prob > F	0.002			<0.001		

* 0.05 < *p* < 0.01; ** 0.01 < *p* < 0.001. Original variable names: ^#^ fleet size (paratransit)/2006 population aged 65 and over (CSD).

**Table 4:** Association of environmental and individual characteristics with social participation in urban areas.

Variable	Model 1 (*n* = 277)			Model 2 (*n* = 243)		
	exp(b)	exp(b) 95% CI		exp(b)	exp(b) 95% CI	
Paratransit fleet size ^#^ (CSD) (vs. Q1)						
Q2	1.443	0.902	2.309	1.333	0.798	2.228
Q3	0.576 ***	0.483	0.686	0.862	0.607	1.225
Q4	n/a			n/a		
Paratransit fleet size^#^ x social deprivation index (vs. Q1)						
Q2	0.471	0.187	1.188	0.355 *	0.160	0.787
Q3	0.822	0.632	1.069	0.883	0.735	1.061
Q4	n/a			n/a		
Stores (DA) (vs. Q1)						
Q2	1.088	0.837	1.415			
Q3	1.370 *	1.021	1.838			
Q4	n/a			n/a		
Social deprivation index (worst to best) (DA)	1.204 *	1.017	1.425	1.162 *	1.025	1.318
Age				1.011	0.989	1.032
Physical health				1.017	0.923	1.122
Activities of daily living				1.562 ***	1.232	1.981
Positive social interaction				1.042 *	1.010	1.075
Man (vs. woman)				0.737	0.537	1.012
Education (vs. no diploma)						
High school diploma				1.096	0.676	1.775
Postsecondary diploma				1.444 **	1.110	1.880
McFadden’s pseudo R^2^	0.010			0.036		
Prob > F	<0.001			<0.001		

* 0.05 < *p* < 0.01; ** 0.01 < *p* < 0.001; *** *p* < 0.001. n/a: no respondents in the corresponding quartile in this living area. Original variable names: # fleet size (paratransit)/2006 population aged 65 and over (CSD).

**Table 5:** Association of environmental and individual characteristics with social participation in rural areas.

Variable	Model 1 (*n* = 592)			Model 2 (*n* = 518)		
	exp(b)	exp(b) 95% CI		exp(b)	exp(b) 95% CI	
Concentration of older adults ^#^ (vs. Q1)						
Q2	2.430 ***	2.030	2.909	2.370 ***	1.700	3.304
Q3	2.148 ***	1.884	2.448	2.386 ***	1.817	3.133
Q4	n/a			n/a		
Libraries/km^2^ (DA) (vs. Q2) Q3	0.738 *	0.581	0.939	0.707 *	0.545	0.919
At least one leisure resource (DA) (vs. none)	1.205 *	1.018	1.425			
At least one shopping center (DA) (vs. none)	1.535 *	0.998	2.361			
Age				1.011	0.997	1.024
Physical health				1.082	0.990	1.184
Activities of daily living				1.289 **	1.097	1.514
Positive social interaction				1.045 ***	1.021	1.069
Man (vs. woman)				0.924	0.774	1.104
Education (vs. no diploma)						
High school diploma				1.517 **	1.180	1.950
Postsecondary diploma				1.135	0.924	1.394
McFadden’s pseudo R^2^	0.005			0.018		
Prob > F	<0.001			<0.001		

* 0.05 < *p* < 0.01; ** 0.01 < *p* < 0.001; *** *p* < 0.001. n/a: no respondents in the corresponding quartile in this living area. Original variable names: ^#^ 2006 population aged 65 and over (DA)/2006 population aged 65 and over (CSD).

## Supplementary Materials

The following are available online at https://www.mdpi.com/1660-4601/17/22/8399/s1, Table S1. Environmental characteristics classified according to ICF domains.
