# Supplementary material for: Environmental Characteristics Associated with Older Adults’ Social Participation: The Contribution of Sociodemography and Transportation in Metropolitan, Urban, and Rural Areas
Source: Int J Environ Res Public Health. 2020 Nov 13;17(22):8399. doi: 10.3390/ijerph17228399 (PMC7697474; doi:10.3390/ijerph17228399)
Supplement: Supplementary file 1 [file ijerph-17-08399-s001.pdf]

International Journal of *Environmental Research and Public Health*

MDPI

Article

Environmental characteristics associated with older adults' social participation: the contribution of sociodemography and transportation in metropolitan, urban and rural areas

Table S1. Environmental characteristics classified according to ICF domains

Characteristics	Collection scale	Analysis scale	Source	Variable scale	Mean (SD)[a]	Min[a]	Max[a]
**PRODUCTS AND TECHNOLOGY**							
**e120 - Products and technology for personal indoor and outdoor mobility and transportation**							
Subsidized Home Adaptations	CSD	CSD (1,260)	1	Discrete	0.5 (1.6)	0	21
**e140 - Products and technology for culture, recreation and sport**							
Sports centers [b]	DA	DA (13,776); CSD (1,320)	2	Discrete	0.0 (0.2)	0	2
Cultural centers [b]	DA	DA (13,776); CSD (1,320)	2	Discrete	0.0 (0.2)	0	6
**e145 - Products and technology for the practice of religion and spirituality**							
Churches	DA	DA (13,776)	2	Discrete	0.2 (0.8)	0	63
Optometrists [b]	DA	DA (13,776); CSD (1,320)	2	Discrete	0.1 (0.5)	0	31
Drugstores	DA	DA (13,776)	2	Discrete	0.1 (0.4)	0	21
**e160 - Products and technology of land development**							
Land use diversity	DA	DA (13,394)	3	Continuous	0.3 (0.1)	0.0	0.8
Banks	DA	DA (13,776)	2	Discrete	0.2 (1.1)	0	76
Libraries [b]	DA	DA (13,776); CSD (1,320)	2	Discrete	0.0 (0.1)	0	7
Coffee shops	DA	DA (13,776)	2	Discrete	0.1 (0.8)	0	51
Shopping centers [b]	DA	DA (13,776); CSD (1,320)	2	Discrete	0.0 (0.3)	0	12
Convenience stores	DA	DA (13,776)	2	Discrete	0.2 (0.6)	0	40
Grocery stores	DA	DA (13,776)	2	Discrete	0.2 (0.7)	0	50

Hotels [b]	DA	DA (13,776); CSD (1,320)	2	Discrete	0.1 (0.5)	0	23
Hardware stores [b]	DA	DA (13,776); CSD (1,320)	2	Discrete	0.1 (0.3)	0	4
Restaurants	DA	DA (13,776)	2	Discrete	1.0 (3.7)	0	272
Hair salons	DA	DA (13,776)	2	Discrete	0.6 (1.5)	0	101
Gas stations	DA	DA (13,776)	2	Discrete	0.1 (0.4)	0	8
**NATURAL ENVIRONMENT AND HUMAN-MADE CHANGES TO ENVIRONMENT**							
**e215 - Population**							
Population aged 65 and over (%)	DA, CSD	DA (13,776); CSD (1,320)	4	Continuous	0.1 (0.1)	0	1.0
Population concentration (Pop65 DA / pop65 CSD)	DA, CSD	DA (13,776)	4	Continuous	0.1 (0.2)	0	1.0
Material deprivation index [1]	DA	DA (10,178)	5	Continuous	0.0 (0.0)	-0.1	0.2
Social deprivation index [1]	DA	DA (10,178)	5	Continuous	0.0 (0.0)	-0.1	0.1
**e220 - Flora and fauna**							
Greenness (Mean of greenest pixel values 100x100m)	PC	DA (6,657)	6	Continuous	0.5 (0.1)	0.2	0.8
**e225 - Climate**							
Annual average daily maximum temperature (Celsius)	PC	DA (6,657)	7	Continuous	10.2 (1.4)	-4.0	11.7
Annual average daily minimum temperature (Celsius)	PC	DA (6,657)	7	Continuous	0.4 (1.4)	-10.3	2.7
Annual number of rain events	PC	DA (6,657)	7	Discrete	108.7 (6.8)	40	125
Annual average amount of rain per event (mm)	PC	DA (6,657)	7	Continuous	14.9 (2.4)	4.9	22.9
Annual average length of rain events (days)	PC	DA (6,657)	7	Continuous	2.6 (0.2)	2.1	4.7
Annual number of snow events	PC	DA (6,657)	7	Discrete	70.8 (13.2)	59	208

Annual average amount of snow per event (mm)	PC	DA (6,657)	7	Continuous	9.8 (1.1)	3.7	13.1
Annual number of heat event starts based on maximum temperature	PC	DA (6,657)	7	Discrete	1.2 (0.5)	0	4
Annual number of frost days (min temp < 0) (days)	PC	DA (6,657)	7	Discrete	205.4 (16.0)	94	230
**e240 - Light**							
Annual mean nighttime brightness	PC	DA (6,655)	8	Continuous	53.1 (15.4)	0.0	63.0
**SERVICES, SYSTEMS AND POLICIES**							
**e520 - Open space planning services, systems and policies**							
**e540 - Transportation services, systems and policies**							
Roads (km)	DA	DA (13,634); CSD (1,320)	9	Continuous	20.4 (123.3)	0.0	4,172.0
Ridership (public transit) / Pop65*1000	TC	CSD (492)	10, 15	Continuous	700.1 (3,599.4)	0.0	60,247.2
Kilometers served (public transit) / roads (km)	TC	CSD (248)	10, 15	Continuous	3,876.8 (20,010.8)	0.2	241,998.5
Kilometers traveled annually (public transit) / roads (km)	TC	CSD (329)	10, 15	Continuous	467,679.7 (1,193,467.0)	0.0	12,726,308.0
Seniors' reduced fare (public transit)	TC	CSD (372)	10, 15	Binary	0.2 (0.4)	0	1
Ridership (paratransit) / Pop65*1000	TC	CSD (1,029)	10, 14	Continuous	7.8 (35.2)	0.0	793.1
Kilometers traveled (paratransit) / roads (km)	TC	CSD (994)	10, 14	Continuous	29,121.0 (93,751.0)	1.2	1,686,834.0
Fleet size (paratransit) / Pop65	TC	CSD (1,029)	10, 14	Continuous	1.1 (3.6)	0.0	63.5
Road intersection density	DA	DA (13,776)	11	Continuous	46.0 (27.6)	0.0	127.0
Senior vehicle owners (%)	DA	DA (13,041)	12	Continuous	53.2 (0.2)	0.0	100.0

Seniors with a driver's or probationary license	DA	DA (13,105)	12	Continuous	71.6 (0.2)	4.3	100.0
**e535 - Communication services, systems and policies**							
Post offices [b]	DA	DA (13,776); CSD (1,320)	2	Discrete	0.2 (0.4)	0	9
**e545 - Civil protection services, systems and policies**							
Non-violent Crime Severity Index [2]	PS	CSD (1,284)	13	Continuous	82.9 (22.1)	20.7	335.2
Violent Crime Severity Index [2]	PS	CSD (1,284)	13	Continuous	81.3 (46.4)	12.5	878.5
**e580 - Health services, systems and policies**							
Medical clinics [b]	DA	DA (13,776); CSD (1,320)	2	Discrete	0.1 (0.3)	0	13
Dentist offices [b]	DA	DA (13,776); CSD (1,320)	2	Discrete	0.2 (0.8)	0	40

DA: Dissemination area; CSD: Census subdivision; TC: Transit corporation; PS: Police service; PC: Postal code

1. *Société d'habitation du Québec* (2008); 2. DMTI Enhanced Points of Interest (2010); 3. Ministry of Municipal Affairs and Land Occupancy (2013); 4. Canadian Census (2006); 5. *Institut national de santé publique du Québec* (2011); 6. Landsat Normalized Difference Vegetation Index (2009); 7. Canadian Forest Service, Natural Resources Canada (2009); 8. DMSP-OLS Nighttime Lights Time Series (2009); 9. DMTI Route Logistics (2010); 10. Transportation survey (2016); 11. Property assessment (2012); 12. *Société de l'assurance automobile du Québec* (2016); 13. Uniform Crime Reporting Survey (2008); 14. Paratransit statistical directory, Ministry of Transport, Sustainable Mobility and Transport Electrification (2015); 15. Activity report (2009) and bus and metro networks (2015), Montreal Transit Corporation.

1. Pampalon, R.; Hamel, D.; Gamache, P., Health inequalities in urban and rural Canada: Comparing inequalities in survival according to an individual and area-based deprivation index. *Health Place* **2010,** 16, (2), 416-420.
2. Statistics Canada, Uniform Crime Reporting Survey. In Minister of Industry, Ed. Ottawa, Ontario, Canada, 2008.
